# Cavernous Sinus Dural Arteriovenous Fistula Initially Misdiagnosed As Ocular Myasthenia Gravis: A Case Report

**DOI:** 10.7759/cureus.97472

**Published:** 2025-11-21

**Authors:** Shinnosuke Yamamoto, Genya Watanabe, Go Akao, Kenichi Sato, Yasushi Suzuki

**Affiliations:** 1 Department of Clinical Training, National Hospital Organization Sendai Medical Center, Sendai, JPN; 2 Department of Neurology, National Hospital Organization Sendai Medical Center, Sendai, JPN; 3 Department of Neurological Surgery, National Hospital Organization Sendai Medical Center, Sendai, JPN

**Keywords:** cavernous sinus dural arteriovenous fistula, diplopia, edrophonium test, ocular myasthenia gravis, ptosis

## Abstract

Cavernous sinus dural arteriovenous fistulas (CS-DAVF) can mimic ocular myasthenia gravis (OMG), as both may present with diplopia and ptosis. Differentiating between these conditions is critical because misdiagnosis can delay appropriate vascular treatment. Herein, we present the case of a 63-year-old woman who presented with a one-month history of diplopia and left-sided ptosis. Repeated edrophonium tests were positive, leading to an initial diagnosis of OMG. Immunotherapy led to a transient improvement, with decreased Myasthenia Gravis Activities of Daily Living (MG-ADL) and Quantitative Myasthenia Gravis scores. However, persistent diplopia and a new conjunctival injection raised the suspicion of CS-DAVF. Magnetic resonance imaging and angiography revealed enlargement of the left cavernous sinus, while digital subtraction angiography confirmed a left CS-DAVF. This case demonstrates the diagnostic challenges in distinguishing OMG from CS-DAVF. Even when clinical findings and bedside tests strongly suggest OMG, atypical ocular signs, such as conjunctival injection or chemosis, should prompt consideration of CS-DAVF. Careful interpretation of edrophonium results and timely neuroimaging are essential to avoid misdiagnosis.

## Introduction

Cavernous sinus dural arteriovenous fistula (CS-DAVF) is defined as an abnormal arteriovenous shunt in the dura of the cavernous sinus wall [1,2]. CS-DAVF falls within the spectrum of carotid-cavernous fistulas (CCF), which are classified into direct high-flow and indirect low-flow types [3]. Direct CCFs are generally associated with trauma or vessel injury and often present with rapidly evolving, conspicuous symptoms. Conversely, CS-DAVF is regarded as an indirect CCF, typically presenting with a gradual onset and exhibiting variable clinical features depending on the venous drainage pattern. In patients with CS-DAVF, clinical manifestations may include proptosis, chemosis, orbital bruit, diplopia, headache, epiphora, conjunctival injection, and ptosis [1-3].

Ocular myasthenia gravis (OMG) can similarly present with ptosis and diplopia [4]. OMG is a subtype of myasthenia gravis (MG) characterized by weakness limited to the ocular muscles and eyelid function, which worsens with exertion and improves with rest, without generalized involvement. This diagnosis requires several diagnostic modalities, including serologic testing for anti-acetylcholine receptor antibody (AChR-Ab), electrophysiological studies such as repetitive nerve stimulation studies, the edrophonium test to assess responsiveness to cholinesterase inhibitors, and other bedside tests (e.g., the ice pack test) [4]. Only a few case reports have described the difficulty of distinguishing between OMG and CS-DAVF [5-7]. Here, we present the case of a patient diagnosed with OMG based on the Japanese diagnostic criteria and later re-diagnosed with CS-DAVF.

This article was presented orally at the 115th Tohoku Regional Meeting of the Japanese Society of Neurology on September 27, 2025.

## Case presentation

A previously healthy 63-year-old woman presented to our hospital with a one-month history of diplopia and ptosis. Her medical history included hypertension and prior treatment for acute hepatitis at the age of 46 years. Her family and medication history were unremarkable. She initially visited a local ophthalmologist, who performed a positive edrophonium test, raising suspicion of MG. The patient was subsequently referred to our hospital for further evaluation. Physical examination upon admission revealed diplopia on leftward and downward gaze, moderate left-sided ptosis, restricted abduction of the left eye, and a sensation of heaviness in the eyelids upon awakening. Muscle strength in all extremities was within normal limits.

A repeated edrophonium test result was positive, indicating improvements in both diplopia and left-sided ptosis. A repetitive nerve stimulation study at 3 Hz showed no decrease in activity in either the right ulnar or the facial nerves. Chest computed tomography revealed no thymomas or other significant abnormalities. Blood tests for AChR-Ab and anti-muscle-specific tyrosine kinase antibody were negative.

**Figure 1:**
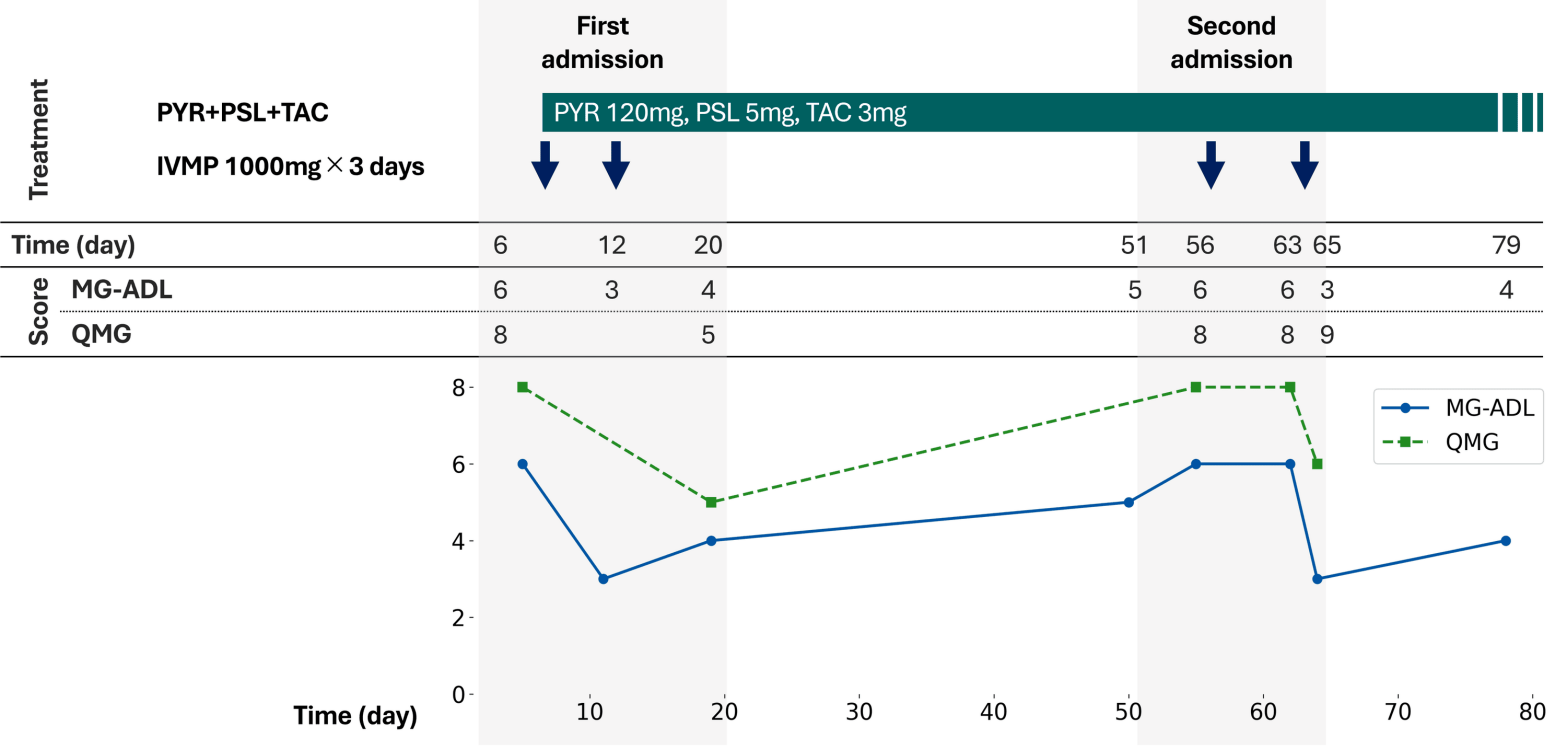
Timeline of the treatment course Days are counted from the date of first hospitalization. PYR: pyridostigmine, PSL: prednisolone, TAC: tacrolimus, IVMP: intravenous methylprednisolone pulse therapy, MG-ADL: Myasthenia Gravis Activities of Daily Living, QMG: Quantitative Myasthenia Gravis

Despite atypical daily symptom fluctuation, characterized by deterioration in the morning and subsequent improvement in the evening, a diagnosis of OMG was established based on clinical findings and Japanese clinical practice guidelines for MG [8]. Subsequently, the patient was administered oral pyridostigmine (120 mg/day), prednisolone (5 mg/day), and tacrolimus (3 mg/day) (Figure 1). She was hospitalized on two occasions, during which treatment with intravenous methylprednisolone (IVMP; 1000 mg/day for three days) was administered in two courses per admission for a total of four courses. After each admission, improvements in ptosis and diplopia were observed in both the patient reports and physician examinations. Following a second admission to our hospital, the patient was transferred to an outpatient clinic. Throughout the clinical course, disease severity and therapeutic response were regularly assessed using the Myasthenia Gravis Activities of Daily Living (MG-ADL) scale [9] and the Quantitative Myasthenia Gravis (QMG) score [10].

**Figure 2:**
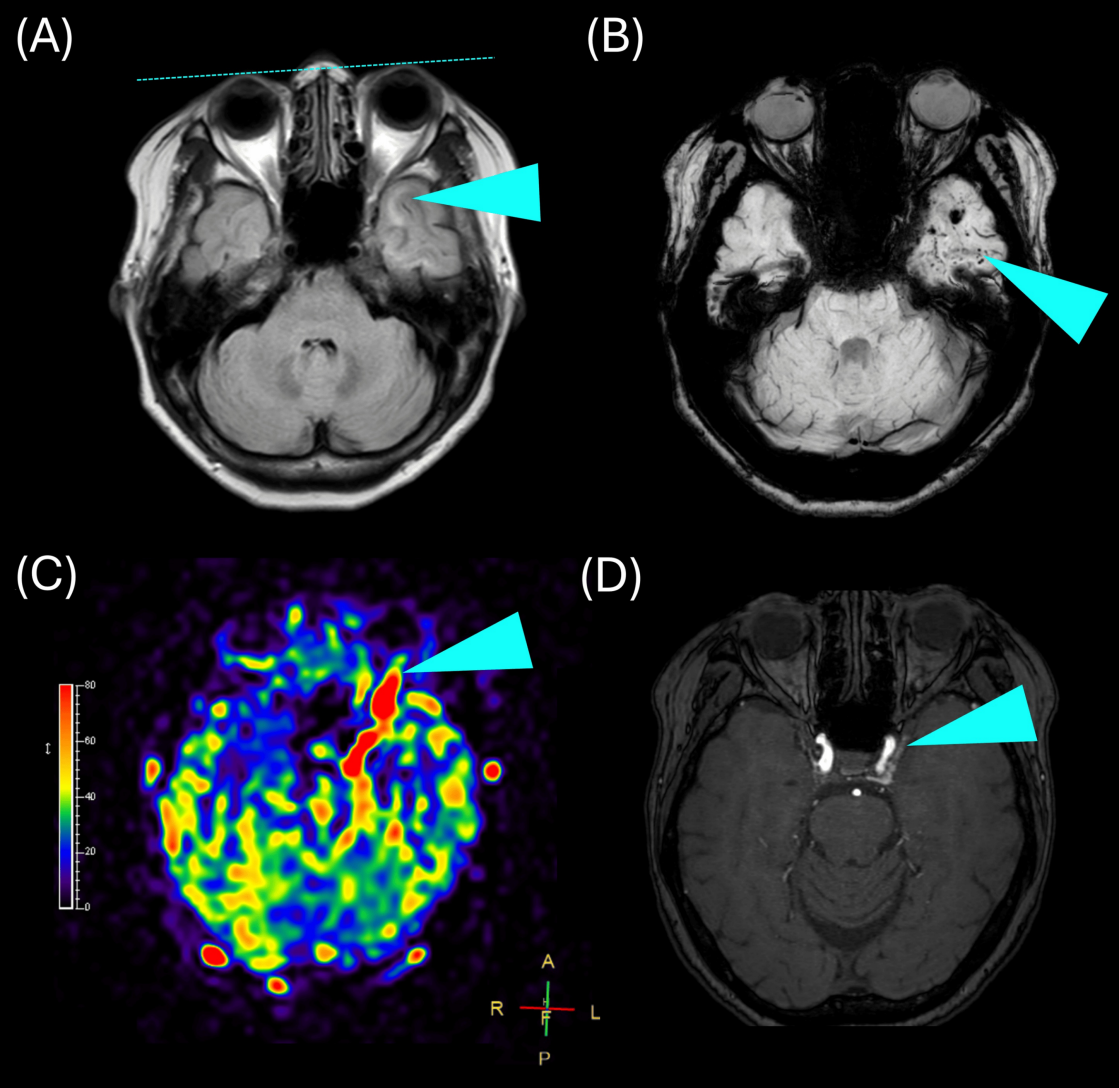
MRI and MRA findings of the left CS-DAVF (A) FLAIR image showing subcortical white matter edema in the left cerebral hemisphere. (B) SWI demonstrating numerous punctate microhemorrhages in the left subcortical white matter. (C) ASL image indicating increased blood flow extending from the left orbit to the parasellar region. (D) MRA clearly visualizing the enlarged left cavernous sinus with prominent internal flow voids. MRI: magnetic resonance imaging, MRA: magnetic resonance angiography, CS-DAVF: cavernous sinus dural arteriovenous fistula, FLAIR: fluid-attenuated inversion recovery, SWI: susceptibility-weighted imaging, ASL: arterial spin labeling

However, two weeks later, she reported persistent diplopia on the left side, resulting in a third admission and a fifth course of IVMP. On this admission, the patient reported a cloudy gelatinous film in her left eye upon awakening in the morning. Physical examination conducted during the third admission revealed worsening of the conjunctival injection, which, in retrospect, had been present to a mild degree since the second admission. No pulsatile bruit or visible proptosis was detected in the upper lateral region of the left eye. Both pupils were equally reactive to light. Given the manifestation of these novel symptoms, CS-DAVF was considered in the differential diagnosis. On MRI, the left cavernous sinus was enlarged compared with the contralateral side, with prominent internal flow voids. Mild proptosis was observed in the left eye (Figure 2). Fluid-attenuated inversion recovery (FLAIR) imaging revealed subcortical white matter edema in the left hemisphere (Figure 2A). Susceptibility-weighted imaging (SWI) revealed numerous punctate microhemorrhages in the left subcortical white matter (Figure 2B). Arterial spin labeling (ASL) demonstrated increased blood flow from the left orbit to the parasellar region (Figure 2C). Magnetic resonance angiography (MRA) provided clear visualization of the left CS (Figure 2D).

**Figure 3:**
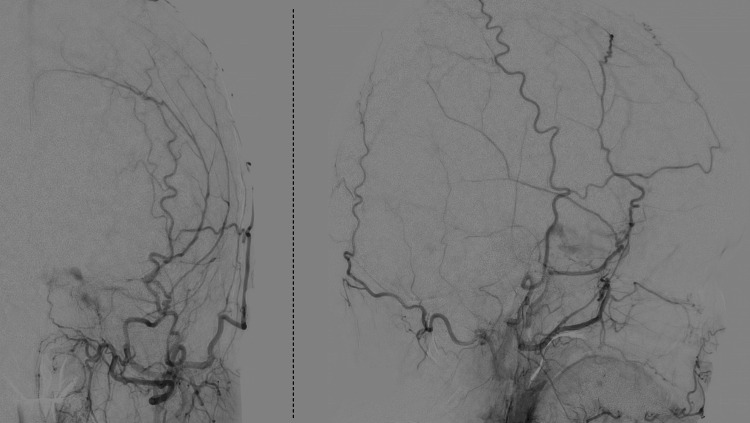
DSA findings of the left CS-DAVF during external carotid artery injection DSA demonstrating early venous filling of the cavernous sinus via dural branches originating from the left external carotid artery, confirming the presence of CS-DAVF. DSA: digital subtraction angiography, CS-DAVF: cavernous sinus dural arteriovenous fistula

Subsequent digital subtraction angiography (DSA) conducted in consultation with a neurosurgeon revealed early venous filling of the cavernous sinus via the dural branches arising from the left external carotid artery, thereby confirming the diagnosis of CS-DAVF (Figure 3). Because cortical venous reflux and a sense of fullness developed in the left ear, endovascular treatment was performed.

Fifty-three days after endovascular treatment, the third edrophonium test was negative. The patient's left conjunctival injection and sensation of fullness in the left ear improved; however, the diplopia and eyelid ptosis persisted. A second DSA was performed, and the neurosurgeon confirmed complete obliteration of the shunt.

## Discussion

This case, ultimately diagnosed as CS-DAVF, necessitated differentiation from OMG. The initial presentation, edrophonium test results, and clinical course were highly consistent with OMG, although certain atypical features complicated the diagnostic process. This case suggests that a CS-DAVF should be considered in the differential diagnosis of OMG.

CS-DAVF and OMG can share overlapping clinical features, particularly diplopia and ptosis, which can make differential diagnosis challenging. CS-DAVF is characterized by an abnormal arteriovenous shunt in the dura of the cavernous sinus wall [1,2]. This condition presents with a wide spectrum of signs and symptoms, including proptosis, chemosis, orbital bruit, diplopia, headache, epiphora, conjunctival injection, and ptosis [1-3]. In contrast, MG is an autoimmune disease that impairs neuromuscular transmission by targeting the neuromuscular junction [11]. OMG usually causes fluctuating diplopia and ptosis and may either remain limited to the eyes or progress to generalized MG. In its generalized form, MG can affect the bulbar muscles (leading to difficulty in swallowing and speaking) as well as the muscles of the limbs, neck, and those used for breathing.

Although both conditions share core symptoms, such as diplopia and ptosis, additional findings, such as chemosis, epiphora, and conjunctival injection in CS-DAVF, helped guide the diagnosis in the present case. Although conjunctival injection is a relatively nonspecific finding, it is readily recognizable both subjectively and objectively. It may therefore serve as an essential clinical clue for differentiating similar cases at an early stage. In the literature, several cases initially diagnosed as OMG were ultimately identified as CCF (Table 1). Accordingly, in our review, we extended the scope to include CCF, as indirect CCF shares substantial overlap with CS-DAVF in terms of terminology and clinical manifestations. In these cases, clinical features typical of OMG were observed, including diurnal symptom fluctuations [5], a positive ice-pack test with symptomatic improvement and responsiveness to MG-directed therapy [6], and AChR-Ab seropositivity [7]. These findings are critical for establishing a clinical diagnosis of OMG.

**Table 1:** Summary of the clinical characteristics of reported cases of CS-DAVF/CCF resembling MG and in the present case (+) indicates the presence of the feature, finding, or response; (-) indicates its absence. MG: myasthenia gravis, IVMP: intravenous methylprednisolone, CCF: carotid–cavernous fistula, CS-DAVF: cavernous sinus dural arteriovenous fistula, ND: not described

Case	Age/sex						Response to MG therapy	Finally diagnosis	Diagnostic pitfall
		Diplopia	Ptosis	Others findings	Diurnal fluctuation	Generalized symptoms			
Eswar et al., 2014 [5]	69M	(+)	Left(+)	Tearing; sense of discomfort behind the left eye; left abducens nerve palsy	(+)	ND	ND	Left CCF	Diurnal fluctuation (+); tearing and discomfort behind the eye, misinterpreted by the patient as seasonal allergic symptoms
Leishangthem and Satti, 2017 [6]	71F	Left(+)	Left(+)	Mydriasis; partial oculomotor and trochlear nerve palsy	ND	(-)	Oral therapy + surgery→(+)	Left CCF	Ice-pack test (+); response to MG therapy (+)
Bireley et al., 2023 [7]	67F	(+)	Right(+)	Sense of discomfort behind the right eye; conjunctival injection (bilateral); tearing; left abducens nerve palsy	(-)	(-)	Oral therapy →(-)	Right CCF	Anti-AChR antibody (+)
Present case	63F	(+)	Left(+)	Left abducens nerve palsy	Atypical	(-)	Oral therapy ＋IVMP → (+)	Left CS-DAVF	Edrophonium test (+); response to MG therapy (+)

At presentation, the patient exhibited the typical features of OMG, resulting in a diagnosis. One of the key reasons for this initial diagnosis was that both the ophthalmologist and neurologist independently observed positive results in two consecutive edrophonium tests in accordance with current diagnostic guidelines. OMG treatment improved subjective symptoms and objective scores (MG-ADL and QMG) (Figure 1). Subsequently, the development of chemosis, epiphora, and new conjunctival injections raised concerns about CS-DAVF, and DSA confirmed its presence.

The edrophonium test is a functional bedside test for diagnosing OMG, with reported sensitivities of 92% to 95.4% and a specificity of 97% [12,13]. Furthermore, despite the ambiguity surrounding the specific mechanism, it has been proposed that transient reactions to cholinesterase inhibitors may not necessarily occur exclusively in diseases of the neuromuscular junction [6]. However, epidemiological studies evaluating the sensitivity and specificity of the edrophonium test are limited; therefore, the findings should be interpreted carefully. Misdiagnosis of CS-DAVF is a significant issue, as OMG treatment could expose patients to unnecessary corticosteroids or thymectomy and critically delay appropriate CS-DAVF treatment.

After endovascular treatment, complete obliteration of the shunt was confirmed using a second DSA. However, the diplopia and ptosis persisted. It may take several months for patients to recover their normal neurological function. If symptoms fail to improve after several months, the possibility of coexisting CS-DAVF and OMG should be considered. To the best of our knowledge, no case combining both diagnoses has been reported. Indeed, it was reported that the mean incidence of DAVF and the mean prevalence of MG were 0.29 cases per 100,000 person-years [14] and 173.3 cases per million (95% CI: 129.7-215.5) [15], respectively, highlighting the low frequency of both of these conditions, CS-DAVF and MG. However, since the possibility of coexistence cannot be completely ruled out, careful follow-up is strongly recommended.

## Conclusions

This case report describes a patient with a clinical presentation initially suggestive of OMG. This case underscores the need to include CS-DAVF in the differential diagnosis, particularly in cases of unilateral ocular muscle involvement, even when patients meet typical clinical criteria and exhibit significant findings consistent with OMG. Moreover, although the edrophonium test is recognized for its high sensitivity and specificity, caution must be exercised when interpreting its results in clinical practice.
